# Isolated human uterine telocytes: immunocytochemistry and electrophysiology of T-type calcium channels

**DOI:** 10.1007/s00418-014-1268-0

**Published:** 2014-09-12

**Authors:** Sanda Maria Cretoiu, Beatrice Mihaela Radu, Adela Banciu, Daniel Dumitru Banciu, Dragos Cretoiu, Laura Cristina Ceafalan, Laurentiu Mircea Popescu

**Affiliations:** 1 Division of Cell Biology and Histology, Carol Davila University of Medicine and Pharmacy, 050474 Bucharest, Romania; 2 Department of Ultrastructural Pathology, Victor Babeş National Institute of Pathology, 050096 Bucharest, Romania; 3 Department of Neurological and Movement Sciences, University of Verona, 37134 Verona, Italy; 4 Department of Anatomy, Animal Physiology and Biophysics, Faculty of Biology, University of Bucharest, 050095 Bucharest, Romania; 5 Department of Molecular Medicine, Victor Babeş National Institute of Pathology, 050096 Bucharest, Romania; 6 Division of Advanced Studies, Victor Babeş National Institute of Pathology, 050096 Bucharest, Romania

**Keywords:** Telocytes, Human myometrium, T-type calcium channels, Ca_V_3.1, Ca_V_3.2

## Abstract

Recently, telocytes (TCs) were described as a new cell type in the interstitial space of many organs, including myometrium. TCs are cells with very long, distinctive extensions named telopodes (Tps). It is suggested that TCs play a major role in intercellular signaling, as well as in morphogenesis, especially in morphogenetic bioelectrical signaling. However, TC plasma membrane is yet unexplored regarding the presence and activity of ion channels and pumps. Here, we used a combination of in vitro immunofluorescence and patch-clamp technique to characterize T-type calcium channels in TCs. Myometrial TCs were identified in cell culture (non-pregnant and pregnant myometrium) as cells having very long Tps and which were positive for CD34 and platelet-derived growth factor receptor-α. Immunofluorescence analysis of the subfamily of T-type (transient) calcium channels Ca_V_3.1 and Ca_V_3.2 presence revealed the expression of these ion channels on the cell body and Tps of non-pregnant and pregnant myometrium TCs. The expression in TCs from the non-pregnant myometrium is less intense, being confined to the cell body for Ca_V_3.2, while Ca_V_3.1 was expressed both on the cell body and in Tps. Moreover, the presence of T-type calcium channels in TCs from non-pregnant myometrium is also confirmed by applying brief ramp depolarization protocols. In conclusion, our results show that T-type calcium channels are present in TCs from human myometrium and could participate in the generation of endogenous bioelectric signals responsible for the regulation of the surrounding cell behavior, during pregnancy and labor.

## Introduction

Regulation of contractile activity of the uterus is an important process, and numerous studies aimed to determine the mechanism of uterine activation during term and preterm parturition (Aguilar and Mitchell 2010). Hitherto, little is known about the physiological triggers of uterine contraction, and the roles of interstitial cells and matrix are frequently neglected (Hutchings et al. 2009). However, there are some new cellular elements in the interstitial space—the telocytes (TCs)—that seem to be important (jointly with other connective tissue cells) for the normal functionality of mammalian organs (Zheng et al. 2012, 2013; Cretoiu et al. 2012a; Mou et al. 2013; Corradi et al. 2013; Díaz-Flores et al. 2013; Matyja et al. 2013; Luesma et al. 2013) including human uterus (Popescu et al. 2006b, 2007; Cretoiu et al. 2011). TCs have a small cell body with very long distinctive prolongations—the telopodes (Tps) (Popescu and Faussone-Pellegrini 2010). A TC can have one up to five Tps with alternating podoms (dilated segments) and podomers (thin segments). Podoms offer accommodation for mitochondria, ER and caveolae, a trio involved in calcium homeostasis (Gherghiceanu and Popescu 2012). Tps have a dichotomous branching pattern, building a 3D network due to homocellular junctions (Ceafalan et al. 2012; Cretoiu et al. 2012b), and occupy a strategic position in relation to stem cell niches, blood capillaries and/or nerve bundles (Gherghiceanu and Popescu 2011; Popescu and Nicolescu 2013). Heterocellular nanocontacts were frequently described between TCs and myocytes (Gherghiceanu and Popescu 2011; Cretoiu et al. 2012a) or TCs and immune cells (Popescu et al. 2005). TCs were shown to release ectosomes and/or exosomes suggesting a role in intercellular signaling (Cretoiu et al. 2013). The immunohistochemical profile of TCs is not yet defined by specific markers; however, several studies claim that TCs are frequently found to be positive for CD34 and PDGFRα or PDGFRβ within the connective tissue in mucosa and submucosa of different organs or in the interstitium of the muscular coat of cavitary organs (Vannucchi et al. 2013; Chen et al. 2013; Milia et al. 2013; Xiao et al. 2013), as well as for Oct4 in mouse lung (Galiger et al. 2014). The gene profile of TCs was reported (Zheng et al. 2013) in mouse lung, as well as patterns of mouse TC-specific gene profiles on chromosome 1 (Sun et al. 2014). The TCs proteome certified that indeed, TCs are different from fibroblasts and from endothelial cells (Zheng et al. 2014a, b).

Electrophysiologically, there is evidence about the fact that atrial and ventricular TCs present large conductance Ca^2+^-activated K^+^ currents and inwardly rectifying K^+^ currents, but not transient outward K^+^-currents and ATP-sensitive potassium currents (Sheng et al. 2014). Moreover, in human uterus, small-conductance calcium-activated potassium currents have been detected (Rosenbaum et al. 2012), and preliminary in vitro electrophysiological evaluation of the TCs in non-pregnant uterus revealed a hyperpolarization-activated chloride inward current with calcium dependence (Cretoiu et al. 2013).

The subfamily of T-type (transient) calcium channels consists of several different subunits: α_1_, α_2_δ, β_1–4_ and γ. Differences in molecular structure of α_1_ subunits subdivided them as Ca_V_3.1 (α_1G_), Ca_V_3.2 (α_1H_) and Ca_V_3.3 (α_1I_). These subunits are most often found in cells that have pacemaker activity (Blanks et al. 2007; Senadheera et al. 2013). Although T-type calcium channels have been extensively studied in human myometrium (Young et al. 1993; Young and Zhang 2005), no evidence was yet documented in uterine TCs.

Our purpose was to determine whether such T-type channels are present in TCs and to study whether there are any differences between the TCs in non-pregnant and pregnant myometrium. Ca_V_3.1 and Ca_V_3.2 were expressed in TCs, on the cell body and in Tps, and found to be less intense in TCs in non-pregnant myometrium. This study provides direct immunofluorescence and electrophysiological evidence for the existence of T-type calcium channels in TCs from human myometrium.

## Materials and methods

### Human tissue samples

Biopsies of human myometrium were obtained from two groups of women (*n* = 8, in each group) under non-pregnant and pregnant state. All tissue samples were obtained in accordance with a protocol approved by the local Bioethics Committee of the University of Medicine and Pharmacy, Bucharest, in accordance with The Code of Ethics of the Declaration of Helsinki. Informed written consent was received from all patients donating tissue samples. None of the subjects were under regular medication for chronic diseases. Non-pregnant myometrium strips were removed from the uterine supraisthmic region from hysterectomy specimens (benign conditions) of premenopausal women. Small strips of pregnant myometrium (between 38 and 40 weeks of gestation) were taken during Caesarean sections, from the upper margin (in the midline) of the lower segment transverse incision.

### Cell cultures

Human myometrial samples were collected into sterile tubes containing Dulbecco’s Modified Eagle’s Medium (DMEM), supplemented with fetal bovine serum (FBS) 2 %, HEPES (1.5 mM) as well as 200 IU/ml penicillin, 200 UI/ml streptomycin and 0.50 µg/ml amphotericin B (Fungizone) (all from Gibco/Life Technologies Ltd., Paisley, UK), placed on ice and transported to the cell culture laboratory. Samples were processed within 30 min from surgery and rinsed with sterile DMEM. The myometrial samples were dissected under a stereomicroscope and minced into small pieces of about 1 mm^3^, subsequently washed and incubated with gentle agitation for 30 min, at 37 °C, with collagenase Ia (Sigma-Aldrich, St. Louis, MO, USA) 10 mg/ml and DNAase I (0.1 nm/mg) in DMEM supplemented with FBS 10 %, HEPES 1.5 mM, 100 IU/ml penicillin, 100 UI/ml streptomycin and 0.25 µg/ml fungizone. The dispersed cells were separated from non-digested tissue by filtration through a cell strainer (100 mm), collected by centrifugation of the filtrate at 250 g for 10 min, at room temperature (22 °C) and suspended in culture medium. Cells were distributed in 25-cm^2^ plastic culture flasks (BD Falcon, San Jose, CA, USA) or on glass coverslips into 24-well plates (BD Labware, San Jose, CA, USA) at a density of 5 × 10^4^ cells/cm^2^. Medium was changed every 48 h. Cells were maintained at 37 °C in a humidified atmosphere (5 % CO_2_ in air) until becoming semi-confluent (usually 4 days after plating) when the cells were detached using 0.25 % trypsin and 2 mM EDTA and replated at the same density of 5 × 10^4^ cells/cm^2^. Experiments were performed between passages 1 and 4. Cells were examined and photographed under a Nikon inverted TE200 microscope equipped with a Nikon DN-5 digital camera.

### Immunocytochemistry

To visualize cell expressing T-type calcium channels, immunofluorescent labeling was performed on cells grown on coverslips (Ciontea et al. 2005). Cell cultures were obtained from both non-pregnant and pregnant myometrium. Samples were fixed in 2 % paraformaldehyde for 10 min, washed in PBS and then incubated in PBS containing 2 % bovine serum albumin (BSA) for another 10 min. Next, cells were washed and permeabilized with 0,075 % saponin in PBS for 10 min (all reagents from Sigma-Aldrich, St. Louis, MO, USA). Incubation with the primary antibodies was performed at room temperature for 1 h using antihuman antibodies with the listed specificities and working dilutions (Table 1). Primary antibodies were detected with secondary anti-mouse antibody conjugated to AlexaFluor 546, 1:300 or AlexaFluor488, 1:300; secondary goat anti-rabbit antibody conjugated to AlexaFluor 488, 1:250; donkey anti-goat antibody conjugated to AlexaFluor 546, 1:250 all from Invitrogen Molecular Probes, Eugene, OR, USA. Nuclei were finally counterstained with 1 µg/ml 4′,6-diamidino-2-phenylindole (DAPI) (Sigma-Aldrich). Negative controls were obtained following the same protocol, but omitting the primary antibodies. Samples were examined under a Nikon TE300 microscope equipped with a Nikon DS-Qi1 camera, Nikon PlanApo 20× and 40× objectives, and the appropriate fluorescence filters. The immunolabelled samples were photographed, and images were randomly selected and evaluated for fluorescent intensity analysis. The images were loaded into Image J, the region of interest (ROI) traced and the ‘gray level intensity’ analyzed. Background gray level intensity was also measured and subtracted for each image (Burgess et al. 2010; Radu et al. 2014). Resulting data were statistically analyzed using Microsoft Excel analysis tool pack.

**Table 1:** Panel of antibodies

Antibody	Code no.	Host species	Dilution
T-type Ca^2+^ CP α1H	sc-377510	Mouse	1:150
T-type Ca^2+^ CP α1G	sc-28617	Rabbit	1:200
CD34	sc-7045	Goat	1:50
Smooth muscle actin Ab-1	MS-113-P	Mouse	1:200
PDGFRα	sc-338	Rabbit	1:100

### Patch-clamp recordings

TCs from non-pregnant uterus were recorded in whole-cell configuration under the voltage-clamp mode, using an AxonPatch 200B amplifier (Molecular Devices, USA). Electrodes were pulled from borosilicate glass capillaries (GC150F; Harvard Apparatus, Edenbridge, Kent, UK) and heat polished. The final resistance of the pipette, when filled with internal solution, was 3–4 MΩ. The perfusion was performed with an MPS-2 (World Precision Instruments, Sarasota, FL, USA) system, with the tip placed at approximately 100 μm from the cell. Membrane currents were low-pass filtered at 3 kHz (−3 dB, 3 pole Bessel) and sampled with a Axon Digidata 1440 data acquisition system (Molecular Devices, USA) using pClamp 10 software in gap-free mode. All electrophysiological experiments were performed at room temperature (25 °C).

The bath solution contained (mM): tetraethylammonium (TEA)-Cl 130, BaCl_2_ 10, MgCl_2_ 1, HEPES 10, glucose 10, adjusted to pH 7.4 at 25 °C with TEA-OH. The pipette solution contained (mM): CsCl 137; MgCl_2_ 1; HEPES 10; 1,2-bis(2-aminophenoxy)ethane-*N*,*N*,*N*′,*N*′-tetraacetic acid (BAPTA) 10; Mg-ATP 1, adjusted to pH 7.3 at 25 °C with CsOH. Solutions for T-type calcium channel recordings have been used as previously described (Comunanza et al. 2011), and only EGTA was replaced by BAPTA in the pipette filling solution in order to increase the stability of the baseline.

Two voltage protocols have been applied on TCs from non-pregnant uterus in order to evoke T-type calcium currents: (1) step depolarization protocol with pulses (from −90 to +40 mV) of 100 ms duration, 10-mV increment from a holding potential of −110 mV as previously described (Ohkubo et al. 2005) and (2) brief depolarizing ramp protocol from −90 to +60 mV with a duration of 100 ms (1.5 V/s), as previously described (Comunanza et al. 2011). Mibefradil (1 μM), a potent antagonist for T-type calcium channels (Clozel et al. 1997; Leuranguer et al. 2001), was applied 4 min before and during the brief depolarization ramp protocols.

In order to test whether the calcium current has a steady-state component, we have applied ramp depolarization protocols with different slopes 0.5 and 0.05 V/s, in a similar manner as the protocol previously described (Baruscotti et al. 2000).

Data were analyzed in Clampfit 10.2 (Molecular Devices Corporation, USA) and plotted in Origin 8.6. Statistical analysis was done by paired Student’s *t* test, and data are presented as mean ± SD.

## Results

### TCs identification in myometrial cell cultures

**Fig. 1:**
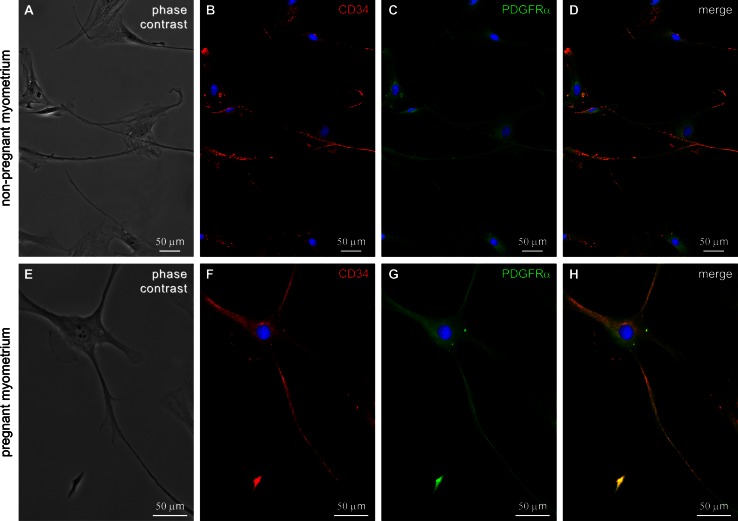
TCs in cell cultures from human non-pregnant/pregnant myometrium after 72 h in culture at first passage. **a**, **e** Phase-contrast microscopy depicts cell morphologies very evocative for TCs: small cell body with very long Tps characterized by a moniliform silhouette (alternation of podoms and podomers). Fluorescence microscopy shows CD34 (**b**, **f**) and PDGFR-α (**c**, **g**)-positive TCs. **d**, **h** Double labeling for both markers showing a different expression pattern for PDGFR-α and CD34 in the same TC. Nuclei were stained with DAPI. *Bar* 50 µm

Under phase-contrast microscopy, in primary cell cultures, TCs were easily distinguished from smooth muscle cells (SMCs) before cell confluence. According to previous studies cell cultures derived from non-pregnant and pregnant myometrium, tissue samples are able to maintain the cell phenotype up to the tenth passage (Leoni et al. 1990; Mosher et al. 2013) and validated primary cell culture usefulness for studying pregnancy and labor (Mosher et al. 2013). In both type of cultures, from non-pregnant and pregnant myometrium, TCs displayed the characteristic silhouette and extend between 1 and 3 long, moniliform Tps (Fig. 1a, e). In order to analyze whether these cells are indeed TCs, we performed immunofluorescence for CD34 and PDGFRα antigens. Immunostaining of non-pregnant and pregnant myometrial cells in culture revealed the presence of TCs as CD34/PDGFRα-positive cells (Fig. 1b, c, f, g), corresponding to the phenotype described by others in situ (Vannucchi et al. 2013; Milia et al. 2013, Xiao et al. 2013; Qi et al. 2012) or in vitro (Mou et al. 2013). Double immunofluorescence staining against CD34 and PDGFRα revealed intense CD34 immunostaining at Tps level, while PDGFRα was expressed mostly in the cell body as it can be observed on merged images (Fig. 1d, h).

### Immunofluorescence for T-type calcium channels in TCs from human myometrium

**Fig. 2:**
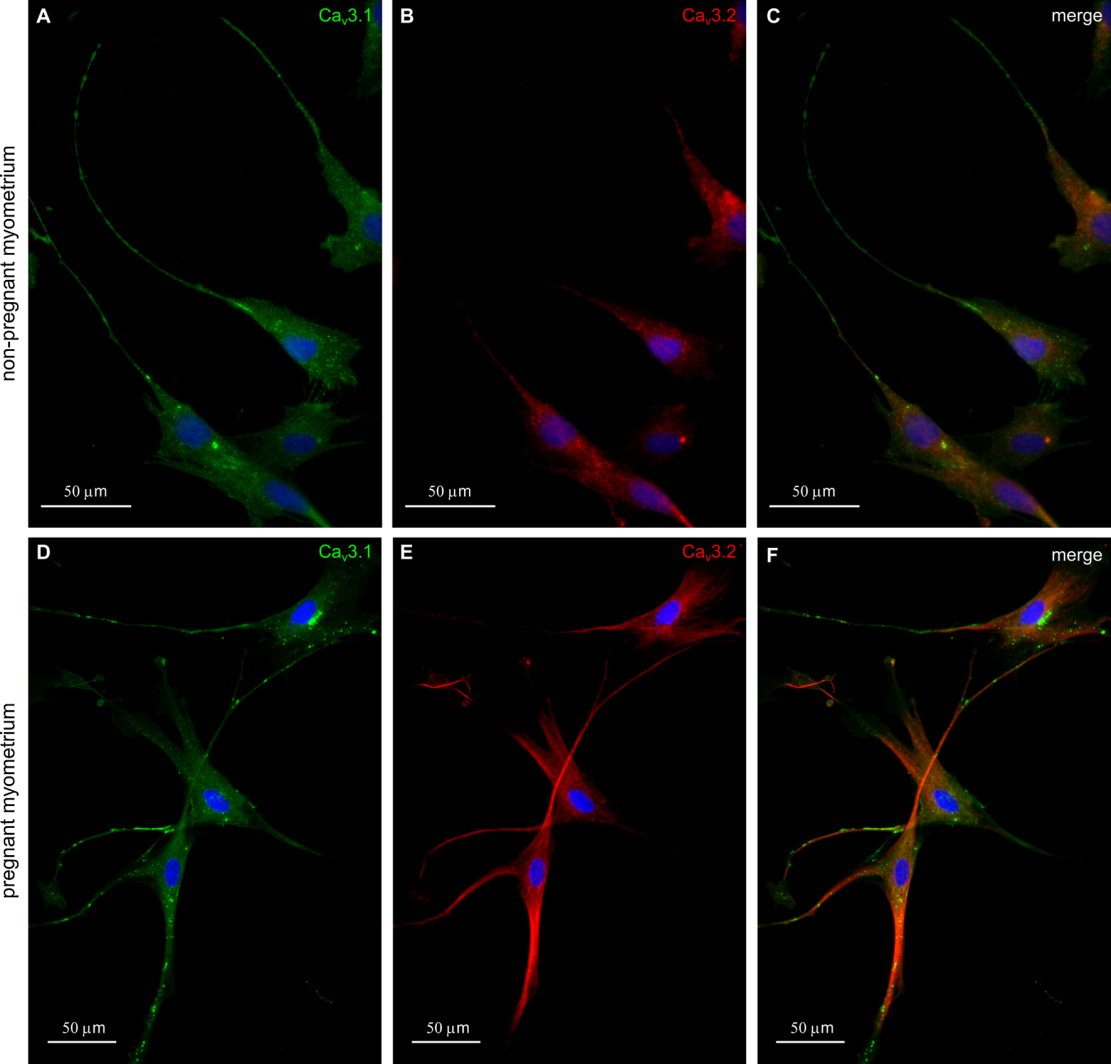
Double immunolabeling for T-type calcium channels in cell cultures from non-pregnant/pregnant myometrium. **a** Ca_V_3.1 immunolabeling (*green*) was detected on TCs cell body, but it was stronger at Tps level, **b** Ca_V_3.2 expression was detected only at cell body level. **c** Co-expression of Ca_V_3.1-positive (*green*) and Ca_V_3.2 (*red*) is presented on merged images. **d** Immunolabeling for T-type calcium channels in cell cultures from pregnant myometrium. Strong staining for Ca_V_3.1 was found in the cytoplasm, adjacent to the nucleus and in the Tps of the TCs. **e** Ca_V_3.2 immunolabeling was found throughout the cytoplasm of TCs and predominantly within Tps. **f** Merged images show co-expression pattern for Ca_V_3.1 (*green*) and Ca_V_3.2 (*red*). Note that both TCs and SMCs (cells with widened cell body without extensions) express Ca_V_3.1 and Ca_V_3.2. Nuclei were stained with DAPI. *Bar* 50 µm

**Fig. 3:**
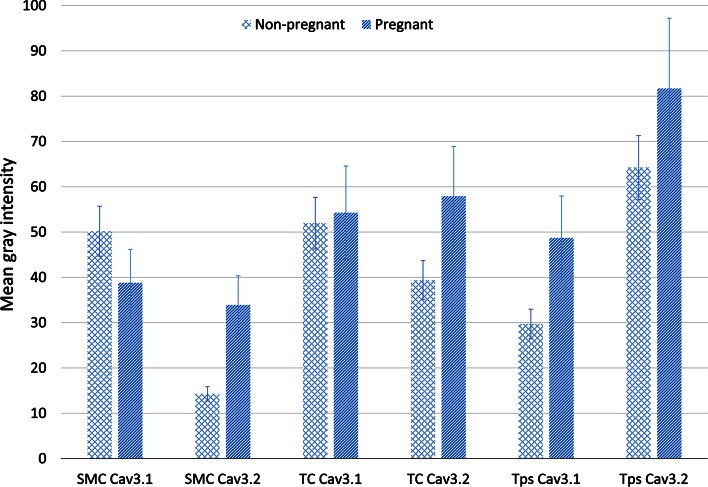
Semi-quantitative analysis of fluorescence intensity of Ca_V_3.1 and Ca_V_3.2 on TCs from non-pregnant and pregnant myometrium. Roughly, one can observe that the level of fluorescence intensity is around 50 or below for SMCs, while in TCs the level is above this value. *Histogram* depicts that Ca_V_3.1 is expressed approximately equal on TCs in non-pregnant versus pregnant myometrium. Instead, Ca_V_3.2 is expressed more in the TCs of the pregnant myometrium versus non-pregnant myometrium. The fluorescence intensity was higher at the level of the Tps for both Ca_V_3.1 and Ca_V_3.2, in non-pregnant and pregnant myometrium

We also investigated the expression of α-subunits Ca_V_3.1 (α_1G_) and Ca_V_3.2 (α_1H_) by immunofluorescence microscopy in cell cultures. Immunohistochemical staining using anti-Ca_V_3.1 and anti-Ca_V_3.2 antibodies showed the expression of α_1G_ and α_1H_ in both non-pregnant and pregnant myometrial cell cultures (Fig. 2). Ca_V_3.1 (α_1G_) and Ca_V_3.2 (α_1H_) reactivity was observed in cells with morphologies suggestive for TCs. In *non*-*pregnant* myometrial cell cultures, T-type voltage-dependent calcium channel Ca_V_3.1 (α_1G_) and Ca_V_3.2 (α_1H_) isoforms revealed differences in their localization: The intensity of Ca_V_3.1 immunostaining was stronger at Tps level (Fig. 2a), while Ca_V_3.2 was expressed only on the cell body (Fig. 2b). Double-labeling immunofluorescence methods were used to provide evidence that both isoforms were expressed in the same TC (Fig. 2c). In *pregnant* myometrial cell cultures, strong staining for Ca_V_3.1 (Fig. 2d) and Ca_V_3.2 (Fig. 2e) was found in the cell body of TCs and in the Tps (Fig. 2f) coexisting in the same cell. We semi-quantitatively evaluated the intensity of the fluorescence for Ca_V_3.1 and Ca_V_3.2 in both SMCs and TCs and also compared the fluorescence intensity in TCs derived from non-pregnant and pregnant myometrium (Fig. 3).

**Fig. 4:**
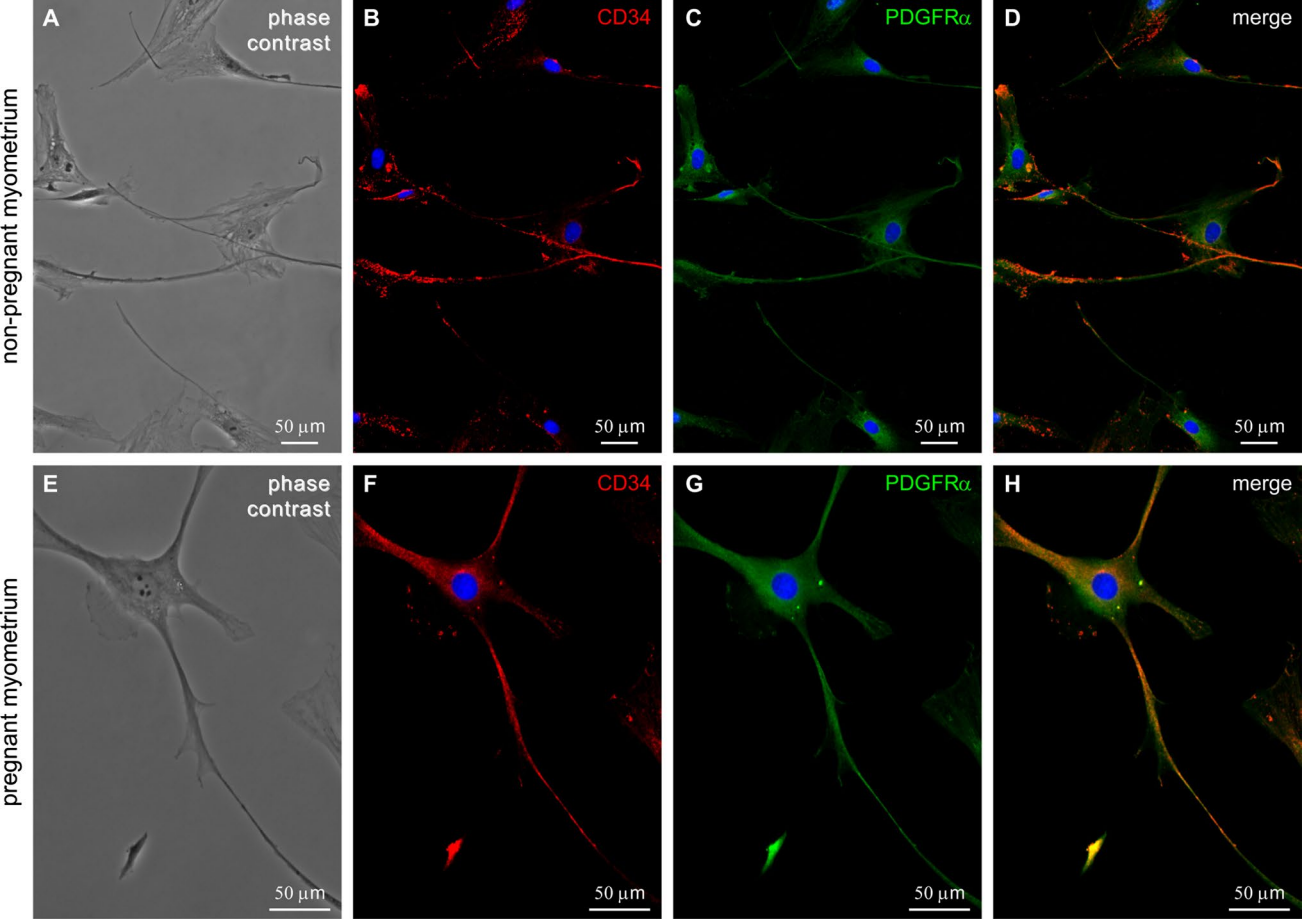
Double immunolabeling for Ca_V_3.1 and α-SMA in cell cultures from non-pregnant/pregnant myometrium. **a**, **e** Phase contrast. **b**, **f** Strong staining for Ca_V_3.1 was found on both cell body and Tps of the TCs. **c**, **g** αSMA immunolabeling was found in SMCs [cells with widened bodies and without cellular extensions (*asterisks*)]. **d**, **h** Merged images of Ca_V_3.1 and α-SMA. Nuclei were stained with DAPI. *Bar* 50 µm

Figure 4 summarizes that at least two types of cells were present in culture: (a) TCs with distinct morphologies and positive for Ca_V_3.1 and (b) SMC with widened cell body without extensions, positive for α-smooth muscle actin (α-SMA). TCs were also positive for α-SMA, but the immunoreactivity was very weak and actin filaments are homogenously distributed and do not form stress fibers like those known to exist in cultured SMCs (Deguchi et al. 2006; Matsumoto and Nagayama 2012). Furthermore, we showed that both cell types (TCs and SMC) were positive for Ca_V_3.1 and Ca_V_3.2. In cell cultures, from both non-pregnant and pregnant myometrium, Ca_V_3.2 expression in TCs was weaker compared with Ca_V_3.1. However, the expression level of Ca_V_3.1 was higher in TCs from pregnant myometrium compared with TCs from non-pregnant myometrium.

### Electrophysiology of T-type calcium channels in TCs from human myometrium

**Fig. 5:**
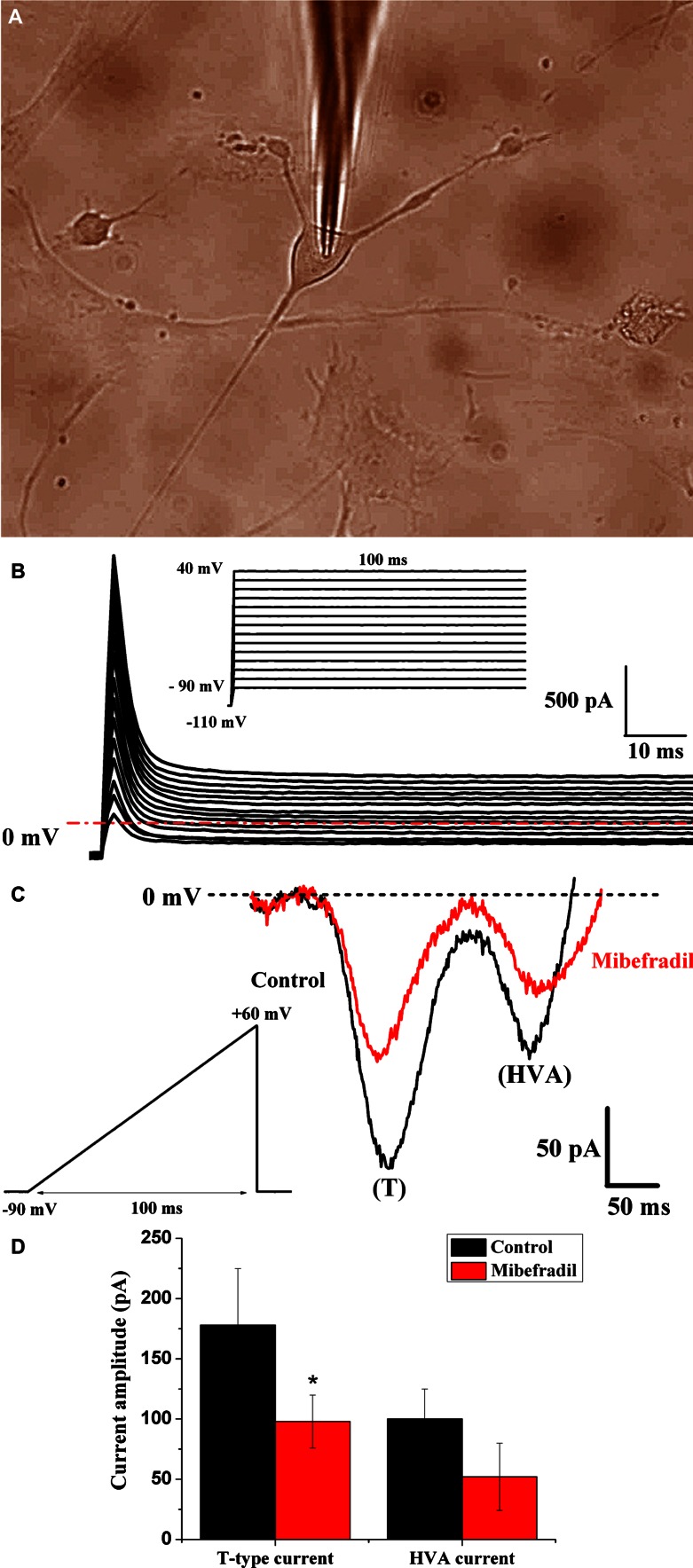
Electrophysiological activity recorded in TCs from non-pregnant myometrium. **a** TC visualized by transmitted light microscopy in phase-contrast illumination mode during patch-clamp recording. **b** TCs generated no detectable voltage-activated calcium currents when tested with a step depolarization protocol. The same protocol was applied on *n* = 12 TCs. *Inset* Step depolarization protocol of 100 ms duration from −90 to +40 mV incremented by 10-mV steps, from a holding potential of −110 mV. **c** Representative T-type calcium current and HVA current in a TC (*black line*) and the blocking effect of 1 μM mibefradil on Ca^2+^ currents (*red line*). *Inset* Ramp commands of 100 ms duration from −90 to +60 mV in voltage-clamp mode. **d** Current amplitude of T-type and HVA calcium currents in control and mibefradil exposure conditions expressed as mean ± SD (*n* = 4)

The presence of T-type calcium channels in TCs was tested by the patch-clamp technique (Fig. 5a) in voltage-clamp mode, using brief ramp depolarization protocol (Fig. 5b, upper insert) and step-depolarizing pulse protocol (Fig. 5c, upper insert).

A standard protocol of step-depolarizing pulses (from −90 to +40 mV) in 10-mV increments of 100 ms duration from a holding potential of −110 mV (Fig. 5b) did not evoke any T-type calcium current neither in TCs (*n* = 12).

 Brief ramp depolarization protocol from −90 to +60 mV with duration of 100 ms evoked two types of calcium currents (Fig. 5c), T-type calcium current [low-voltage activated calcium currents (LVA)] and high-voltage-activated calcium currents (HVA), as previously described by Comunanza et al. (2011). T-type calcium channels were activated at −50 mV. By applying 1 μM mibefradil, we have obtained the amplitude reduction of the T-type calcium current from 178 ± 47 pA (*n* = 4) to 98 ± 22 pA (*n* = 4, *p* < 0.05, paired Student’s *t* test), and of the HVA current from 100 ± 25 pA (*n* = 4) to 57 ± 28 pA (*n* = 4, not significant, paired Student’s *t* test) represented in Fig. 5D. The HVA current was not further characterized. We have obtained an inhibitory effect of mibefradil on HVA currents (e.g., L-type calcium currents), in agreement with previous reports describing mibefradil as a partial antagonist of these channels (Leuranguer et al. 2001).

There is no steady-state component of the calcium currents. Indeed, the currents are abolished at the slowest slope of the ramp depolarization protocol (data not shown).

## Discussion

Human myometrium exhibits in vitro spontaneous contractions which can be influenced by multiple factors (Hutchings et al. 2009; Cretoiu et al. 2011), including the interrelation between SMCs and TCs. Previous studies revealed that TCs can communicate with neighboring muscle cells possibly by gap junctions (Ciontea et al. 2005; Popescu et al. 2006a; Gherghiceanu and Popescu 2011) or by close contacts (Cretoiu et al. 2012a). Recently, it has been suggested that there is a mutual influence between TCs and SMCs acting probably by paracrine mechanisms due to exosome/ectosome release (Cretoiu et al. 2013). However, we assume that, in the myometrium, some other influences could be involved, like, as yet unknown, pathways involving the effects of female sex steroids on the regulation of TCs activity and/or interrelation with SMCs, as suggested by our previous studies (Cretoiu et al. 2006, 2009; Bassotti et al. 2013).

Our study brings evidence on Ca_V_3.1 and Ca_V_3.2 expression in uterine TCs by immunofluorescence staining. Indeed, we have detected the α-subunit of T-type calcium channels in TCs from non-pregnant and pregnant myometrium, on the cell body and in Tps. The expression was less intense in TCs from non-pregnant myometrium. It is an interesting result, as previous immunohistochemistry, qRT-PCR and electrophysiological studies were exclusively dedicated to the Ca_V_3.1 and Ca_V_3.2 expression or T-type currents presence in SMCs from human myometrium (Young et al. 1993; Young and Zhang 2005; Ohkubo et al. 2005; Blanks et al. 2007).

The influence of pregnancy on T-type calcium channel expression in human myometrium is still to be understood. Ca_V_3.1 and Ca_V_3.2 mRNA levels in human myometrial SMCs do not change significantly depending on gestation or labor status (Blanks et al. 2007). On the other hand, different human α_1H_ isoforms resulted from alternative splicing in III-IV linker of Ca_V_3.2 have been predominantly evidenced in non-pregnant uterus with respect to pregnant uterus (Ohkubo et al. 2005). Our study brings the first evidence on Ca_V_3.1 and Ca_V_3.2 expression in TCs from human non-pregnant and pregnant myometrium and highlights differences in localization and level of expression of these proteins due to the pregnancy status.

Further confirmation of T-type calcium channels functionality in TCs from non-pregnant myometrium was done by recording inward currents activated by a brief ramp depolarization protocol that were significantly reduced by mibefradil (1 μM). In fact, mibefradil has been previously described as a strong antagonist of T-type calcium currents (Clozel et al. 1997), which inhibits the bioelectrical signal and uterine contractile forces in human myometrial tissue strips (Young and Zhang 2005).

Previous studies have reported step depolarization protocols as ineffective for activating voltage-gated calcium currents in cardiac progenitor cells (Tufan et al. 2012) or TCs from non-pregnant myometrium (Cretoiu et al. 2013), meanwhile evidencing the presence of these currents by brief ramp depolarization protocols (Tufan et al. 2012). Therefore, the ‘absence’ of inward currents previously reported in interstitial cells from myometrium (Duquette et al. 2005) can be correlated with the use of classical step depolarization protocols.

Differences of electrophysiological parameters (e.g., membrane capacitance, input resistance, membrane resting potential) between uterine TCs and SMCs have been previously reported (Duquette et al. 2005). Compared to the two cellular subpopulations described for uterine SMCs, one population presenting HVA currents and a second population (55 %) having both HVA and LVA currents (Blanks et al. 2007), our study has evidenced that TCs have both types of currents. As the currents in SMCs are evoked by ‘classical’ step depolarization protocols (Blanks et al. 2007), and we show that TCs are responsive only to ramp depolarization protocol and not to step depolarization protocols, it is difficult to exclude the existence of other TCs subpopulations with only HVA currents. Moreover, the steady-state activation/inactivation described for the SMCs population expressing both HVA and LVA currents (Blanks et al. 2007) is absent in TCs presenting both currents.

The presence of T-type calcium channels in uterine TCs might be the missing link for describing the molecular mechanisms by which TCs are involved in mechanical stretching during uterine enlargement in pregnancy. TCs proteomic analysis revealed the up-regulation of myosin-14 (Zheng et al. 2014a) known to be involved in sensory perception (Zong et al. 2012). Periplakin, a protein which links cytoskeleton elements in between and to the junctional complexes, was also found to be upregulated in TCs (Zheng et al. 2014a). Mechanical junctions are indispensable for the proliferation, migration and transformation of different cell types (Leung et al. 2002). Based on previous evidence regarding the existence of heterocellular junctions between TCs and SMC (Cretoiu et al. 2013) and taking into account the existence of cytoskeleton elements (e.g., myosin-14, periplakin), we can hypothesize that TCs could be responsible for detecting the SMC stretch during their enlargement in pregnancy. Moreover, TCs are sometimes described as vimentin-positive cells (Duquette et al. 2005; Zheng et al. 2012), and it was shown that vimentin intermediate filaments determine the mechanical behavior in many cell types (Wang and Stamenovic 2002). Therefore, considering our recent study proving different mechanical reactivity to low-level 1,064-nm laser stimulation of the telopodal lateral extension in non-pregnant versus pregnant myometrium preparations (Campeanu et al. 2014), we propose that TCs play a role of stretch sensor in human myometrium. TCs might be capable of detecting and translate stretch information to the nucleus, determining the production and release of extracellular vesicles and modifying the extracellular homeostasis. These events might also be influenced by steroid hormones, since previous studies showed the existence of estrogen and progesterone receptors in TCs (Cretoiu et al. 2006, 2009). Moreover, we have to emphasize the fact that TCs reactivity to low-level 1,064-nm laser stimulation was influenced by mibefradil (Campeanu et al. 2014) that could imply the contribution of T-type calcium channels in TCs to their capacity as a stretch sensor in human myometrium.

In fetal cardiac myocytes, T-type Ca^2+^ channels were suggested to play an important role in the regulation of cardiomyocyte size. After birth, T-type Ca^2+^ channels are involved in the exit of cardiomyocytes from the cell cycle (Wang et al. 2013). Also, there are suggestive studies about existing linkage between Ca^2+^ influx through T-type Ca^2+^ channels and the cell proliferation (Taylor et al. 2008). In addition, it seems that in vascular smooth muscle cells, T-type channels might ensure a feedback mechanism to hold off excessive vasoconstriction, being versatile players especially in pathophysiological situations (Kuo et al. 2014). Therefore, there is a possibility that T-type Ca^2+^ channels could be only transiently present in TCs. They could influence the regulation of myometrial tissue remodeling—hyperplasia in the first phase and stretch-induced myometrial hypertrophy accompanied by lack of uterine contractions during the second half of pregnancy. Our hypothesis is in concordance with recent data showing that TCs are involved in tissue morphogenesis during development (Zheng et al. 2014a) and play a part in regeneration and aging (Zheng et al. 2014b). Edelstein and Smythies explored the possibility that TCs could be players in a model system based on bioelectrical signaling (Edelstein and Smythies 2014), and probably, this could be applied for human uterus. Considering that Ca_V_3 T-type calcium channel isoforms differentially distribute to somatic and dendritic compartments in central neurons (McKay et al. 2006), it is not surprising to observe our quantitative immunofluorescence data pointing out the more prevalent distribution of Ca_V_3.1 and Ca_V_3.2 in the telopodes compared to the cell bodies of TCs. Although we already have found a correlation between the elongation of telopodes lateral extensions and T-type calcium channels in uterine TCs (Campeanu et al. 2014), a determined role of these channels in TCs ‘bioelectrical signaling’ is still to be understood.

For the moment, the subfamily of T-type (transient) calcium channels Ca_V_3.1 and Ca_V_3.2 presence, on the cell body and Tps of TCs in cell cultures obtained from non-pregnant and pregnant myometrium, might represent the starting point to understand the nature of the molecular mechanism responsible for the generation of spontaneous contractions in the myometrium. In our opinion, it is possible that both TCs and SMCs share some of the signaling machinery used for excitation. The differences found in T-type calcium channels subtypes expression in TCs might suggest that T-type Ca^2+^ channels may be involved in the initiation of action potentials in myometrium at TCs level and then continued at SMCs level. Taking into account that in the last 10 years, a plethora of functions was revealed for the T-type Ca^2+^ channels, especially in the regulation of critical body functions, and that these channels are able to interact with other ion channels, membrane proteins or bioactive lipids (Nilius and Carbone 2014), we can hypothesize that the differences in expression might be under the pregnancy special hormonal environment. Therefore, steroid hormones and also oxytocin might influence T-type Ca^2+^ channels higher expression (and not only) in TCs derived from pregnant myometrium, since it is known that TCs have steroid hormone receptors. These might lead to frequent and sustained contractions able to trigger birth. Some other hypothesis might consist of the possibility that the presence of T-type Ca^2+^ channel calcium signals on TCs might contribute to cell migration and proliferation of myometrial tissue (Rodman et al. 2005) and not point to a pacemaker role.

In conclusion, our data provide immunocytochemical and electrophysiological demonstration of Ca_V_3.1 and Ca_V_3.2 channels expression in uterine TCs. Further functional studies are needed to investigate the function of these channels in non-pregnant and pregnant myometrium as well as in pathological uterine conditions.

## Abbreviations

TCs: Telocytes
Tps: Telopodes
SMCs: Smooth muscle cells
PDGFR: Platelet-derived growth factor receptor
ER: Endoplasmic reticulum
α-SMA: Alpha-smooth muscle actin
Ca_V_3.1: α1G subunit of T-type calcium channels
Ca_V_3.2: α1H subunit of T-type calcium channels
LVA: Low-voltage activated currents
HVA: High-voltage activated currents
